# User Perspectives and Psychophysiological Manifestations of Fatigue with Trunk Orthosis for Dystrophinopathy Patients

**DOI:** 10.3390/bioengineering11080780

**Published:** 2024-08-01

**Authors:** Ahmad Zahid Rao, Muhammad Danish Mujib, Muhammad Abul Hasan, Ahmad O. Alokaily, Tayyaba Tahira, Saad Ahmed Qazi

**Affiliations:** 1 Department of Biomedical Engineering, NED University of Engineering and Technology, Karachi 75270, Pakistan; danishmujib@neduet.edu.pk (M.D.M.); abulhasan@neduet.edu.pk (M.A.H.); 2 Neurocomputation Lab, National Center of Artificial Intelligence, Islamabad 75270, Pakistan; saadqazi@neduet.edu.pk; 3 Department of Biomedical Technology, College of Applied Medical Sciences, King Saud University, Riyadh 11362, Saudi Arabia; 4 King Salman Center for Disability Research, Riyadh 11614, Saudi Arabia; 5 Operative Dentistry and Endodontics Department, Dow International Dental College, Dow University of Health Sciences, Karachi 74200, Pakistan; tayyaba.tahira@duhs.edu.pk; 6 Department of Electrical Engineering, NED University of Engineering and Technology, Karachi 75270, Pakistan

**Keywords:** dystrophy, electromyography (EMG), exertion, fatigue, orthosis, trunk, usability

## Abstract

The chair-mounted passive trunk orthosis (CMPTO) is designed to enhance wheelchair safety for individuals with dystrophinopathy during their daily activities. Given the disease’s progressive nature, it is crucial to ensure that assistive devices are carefully evaluated to prevent overexertion. This study aims to assess the CMPTO’s user experience and its impact on fatigue-related psychophysiological measurements. We conducted electromyography (EMG) evaluations of four trunk muscles and assessed perceived exertion using the Borg CR-10 scale in 40 healthy subjects while they performed seated maximal reaching tasks with the CMPTO. Additionally, fifteen dystrophinopathy patients evaluated the CMPTO for usability with the System Usability Scale. Paired *t*-tests were employed to compare the median frequency (MDF) of EMG signals, the Wilcoxon signed-rank test for evaluating exertion, and the Mann–Whitney U test to compare the usability reported by patients to those of healthy subjects. The 4-way ANOVA revealed that MDF patterns were significantly influenced by task orientation for each muscle. The CMPTO did not cause a significant reduction in the MDF. Tasks requiring greater trunk rotation were perceived as more exhaustive. Patients reported acceptable usability with the CMPTO, with scores higher than those of healthy subjects. The CMPTO’s usability was comprehensively evaluated in both healthy subjects and patients with dystrophinopathy. Our findings indicate that the CMPTO can be safely used by individuals with dystrophinopathy as an assistive device to improve seated comfort and functional abilities.

## 1. Introduction

Dystrophinopathies [1,2] are a group of genetic disorders characterized by progressive muscle weakness and degeneration [3]. These conditions result from mutations in genes responsible for the structure and function of muscle fibers. The severity, age of onset, and specific muscles affected can vary widely among the different types of dystrophinopathies [4]. Common symptoms include muscle wasting, difficulty with movement, and the loss of strength, which can lead to significant physical disabilities [5,6,7,8]. While there is currently no cure for dystrophinopathies, treatments focus on managing symptoms, maintaining mobility, and improving the quality of life for those affected [9]. Understanding the impact of these disorders on patients’ daily lives is crucial for developing comprehensive care strategies and support systems.

The quality of life in dystrophinopathy patients is closely related to fatigue [10]. Fatigue, characterized by a reduction in the force-producing capability of skeletal muscles, leads to difficulties for dystrophinopathy patients in performing voluntary movements [11] and maintaining postural stability [4,12]. High levels of fatigue are observed not only during functional activities but also while using assistive devices designed to improve their quality of life [13,14,15]. Therefore, due to the muscle fragility of dystrophinopathy patients, it is crucial that the design of assistive devices considers not only the movements to be supported but also the preservation of muscle function and hence the impact on muscle fatigue [16].

Assistive devices play a crucial role in enhancing the quality of life for individuals with dystrophinopathy by addressing various challenges, including fatigue levels. The methods employed for this purpose include questionnaire-based feedback and evaluating muscle activities. An investigation focused on the effects of dynamic arm support on fatigue in dystrophinopathy patients by utilizing the rating of the perceived exertion scale. It measured how hard the patient feels their body is working during physical activity with and without the arm support. The dynamic arm support is wheelchair-mountable and utilizes elastic bands to apply the assistive force on the forearm. Its joints are aligned with the user’s joints to follow natural movements. It was found that using the dynamic arm support was less fatiguing during upper limb activities [17]. Similarly, other studies on user experience have shown that the use of upper limb assistive devices, such as passive and semi-active arm supports, can lead to improvements in upper limb function and reduce muscular fatigue, especially for moderately to severely impaired patients [18,19]. Passive devices utilize springs or elastic bands to balance the arm, whereas active devices utilize electric or other power sources for their operations.

Several studies used muscle EMG to evaluate assistive devices. The muscle’s EMG signal can be used with various signal processing techniques for the quantification of muscle fatigue [20], including the widely used median frequency that represents the firing rate of motor units. During fatigue, the motor units that were firing at higher frequencies initially may start to fire at lower frequencies or even drop out entirely. This leads to a shift towards lower frequencies in the median frequency measurement and is used to indicate muscle fatigue [21,22,23,24]. One study developed a hand orthosis to assist gripping function [25]. The design comprised wearable flexible finger structures with hydraulic transmission to provide assistive forces to each finger. They used finger extensor and flexor muscles’ EMG to detect movement intention that in effect drives an electrohydraulic system to perform the grip movement. The hand orthosis was found to decrease the muscle load while increasing the grip force in a dystrophinopathy patient. A case study evaluated the masseter muscle activity to monitor the effect of an occlusal splint in a dystrophinopathy patient over six years. The occlusal splint was made of a vacuum-formed thermoplastic material to ensure the contact of the teeth of the mandible and maxilla during bites when eating. The study concluded that the splint reduced muscle fatigue during masticatory movement [26]. Moreover, trunk assistive devices developed to overcome work-related musculoskeletal disorders, arising from repetitive movements, have also used EMG to report a decrease in muscular fatigue [27,28,29]. The design of these devices utilizes elastic elements that act in a similar line of action as the trunk erector spinae muscles. Hence, they transfer the load of the trunk muscles to the shoulders, pelvic girdle, and knees.

The trunk is an important element of the kinematic chain that enables our upper extremities to reach every point in the reachable functional workspace [30]. A balance between spine stability and trunk flexibility is essential for the seated position [31]. Therefore, for people with trunk control impairment, trunk assistive devices are needed that can provide a balance between their trunk stability and the freedom to optimally perform daily activities involving the upper extremities [32]. The use of postural supports with a wheelchair can significantly enhance the posture and arm function of non-ambulant dystrophinopathy patients [33,34]. However, trunk assistive devices that are developed for wheelchair-bound patients such as the chair-mounted passive trunk orthosis (CMPTO) for dystrophinopathy patients [35] and others [36,37,38,39,40] have not been evaluated for fatigue effects despite showing promising results as an assistive device.

The objective of this study was to evaluate the effect of the CMPTO on fatigue during seated reaching tasks. The present study has several strengths and unique features that enhance its originality and relevance for the advancement of assistive technology in dystrophinopathy. Notably, it uniquely focuses on evaluating the fatigue effects of a trunk assistive device (CMPTO) specifically designed for wheelchair-bound dystrophinopathy patients, a novel approach in this field. This study employs a comprehensive methodology involving both healthy participants and dystrophinopathy patients, combining objective measures (EMG median frequency analysis) and subjective measures (Borg CR-10 questionnaire and System Usability Scale) to provide a robust understanding of the device’s impact on muscle fatigue and user experience. By emphasizing daily living activities, this research bridges the gap between laboratory findings and real-world applications, offering practical insights into improving the quality of life for users. The innovative use of EMG analysis allows for the precise measurement of muscle activity, while the patient-centered design principles ensure that the assistive devices support movement and preserve muscle function, addressing the unique needs and challenges of dystrophinopathy patients.

## 2. Materials and Methods

### 2.1. Participants

Forty (40) healthy male subjects, without any physical disability that may hinder their movement, from the university and local community participated in this study. Their demographic data included the following (mean ± standard deviation): age (21.81 ± 3.13 years) and body mass index (22.92 ± 4.68 kg/m^2^). The subjects had right-handed (*n* = 32) and left-handed (*n* = 8) arm dominance. Dystrophinopathy patients were reached through social media posts in disability-related groups and patients’ contacts. Fifteen (15) male patients tested the CMPTO for its usability, and eight (08) agreed to provide feedback. This research complied with the tenets of the Declaration of Helsinki. This experiment was approved by the research ethics committee of NED University of Engineering & Technology, Karachi (ASRB/878). Informed consent was obtained from each participant prior to the commencement of experimental trials.

### 2.2. Experimental Procedure

**Figure 1:**
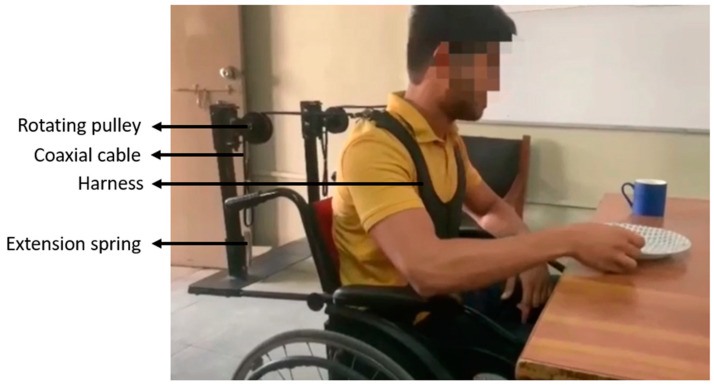
Subject performing activity of daily living using chair-mounted passive trunk orthosis (CMPTO).

This experiment involved the use of the chair-mounted passive trunk orthosis (CMPTO). Figure 1 shows the orthosis being used by a subject. The CMPTO consists of a padded chest harness attached to extension springs at the rear via inelastic cables passing over rotating pulleys. The experiment with healthy subjects comprised two conditions, with orthosis (OR) and without orthosis (noOR). The order of the conditions for each subject was systematically randomized so that half the subjects started the experiment in either condition followed by the other condition. In each condition, the subjects were instructed to perform seated reaching tasks through programmed audios. Each task comprised three stages: ‘Extend’ when the trunk was kept upright with an arm extended along the target direction, ‘Reach’ when the subject had a maximal trunk sway along the target direction with their arm extended, and ‘Return’ when the subject returned to resting position. A 4 s pause was given after the instruction for each of these stages. The targets were placed at five fixed horizontal locations at 0°, 45°, 90°, 135°, and 180° orientations around the subject’s chair. The subjects performed three trials of ten tasks (along five orientations with either upper limb) in each condition. At the end of the experiment, the subjects were asked to fill out the perceived exertion questionnaire.

During testing with dystrophinopathy patients, they were initially asked to perform certain activities to assess their functional capabilities. The instructions were provided in the local language. Thereafter, the caregivers transferred the patients to a wheelchair, where possible, that was pre-fitted with the CMPTO. The caregivers and/or the authors assisted in donning and doffing the harness. The harness was adjusted according to each patient’s comfort. The patients were asked to mimic the activities of daily living for 10 min. Assistance was offered promptly and in accordance with the needs of the individual patients.

### 2.3. Data Measurement

**Figure 2:**
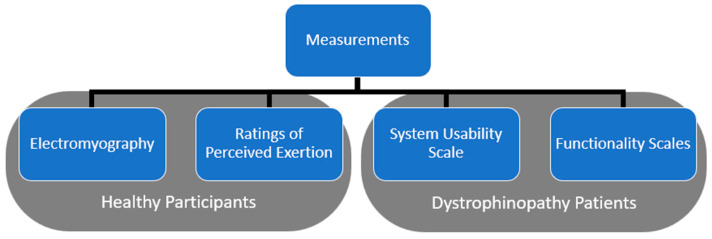
The experimental setup for the healthy participants for reaching tasks.

Figure 2 summarizes the data measured in this experiment. The electromyography and ratings of perceived exertion were measured from healthy participants. The dystrophinopathy patients were assessed for their physical mobility as an indicator of their functionality. They also provided the usability feedback of the CMPTO for the activities of daily living. A description of these measurements follows. 

#### 2.3.1. Electromyography

**Figure 3:**
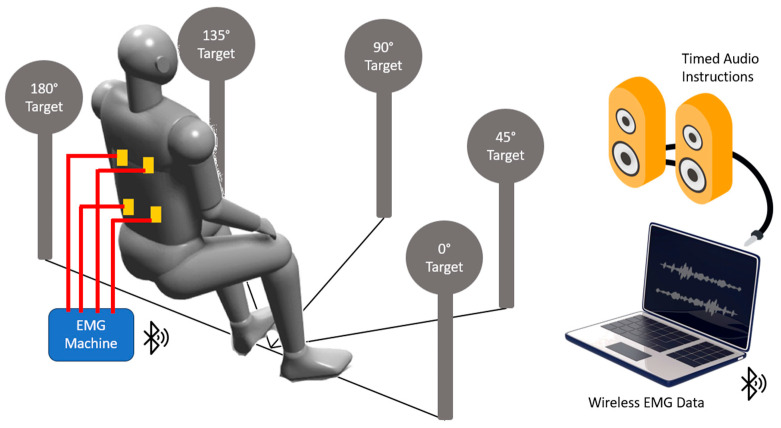
The experimental setup for the healthy participants for reaching tasks. The five target orientations are shown. The timed audio instructions were provided by computer speakers. Four EMG electrodes were placed on participant’s posterior trunk (yellow squares). Wires (red lines) connected the electrodes to the EMG machine. The EMG machine transmitted data to computer via Bluetooth connection.

Figure 3 depicts the experimental setup for measuring the electromyography of the healthy participants. The muscle activity of healthy subjects was recorded using Mobi EMG machine (TMSi, Oldenzaal, The Netherlands) at a sampling rate of 2048 Hz. The system’s high sampling rate ensures the capture of fine details in muscle activity, critical for accurate analysis. The details of the EMG setup include several components such as electrodes, connections, placement locations, equipment positioning, wireless data transfer, and synchronized data acquisition. 

Electrodes and Connections: Low-impedance wires were attached to bipolar surface EMG electrodes, which were placed over four muscle sites: bilateral thoracic erector spinae (RTES, LTES) and lumbar erector spinae (RLES, LLES) muscles. The centers of the bipolar electrodes were placed 5 cm apart, ensuring the proper spatial resolution of the muscle activity. The ground electrode was positioned over the bony prominence of the right iliac crest to reduce noise and improve signal quality. All electrodes and connections adhered to the SENIAM guidelines for placement and setup, ensuring consistency and reliability in the measurements.

Placement Locations: Specific anatomical landmarks were used for electrode placement. TES muscles: 3 cm lateral from the posterior vertical midline at the T9 spinous process level. LES muscles: 3 cm lateral from the posterior vertical midline at the L4 spinous process level.

Equipment Positioning: The portable EMG machine was placed behind the participants at a suitable distance to avoid wire tension and discomfort. The low-impedance wires connected the electrodes to the EMG machine, ensuring stable and high-quality signal transmission.

Wireless Data Transfer: A wireless Bluetooth connection facilitated real-time data transfer between the EMG machine and the computer, allowing for immediate data processing and analysis. This setup minimized the physical constraints on the participants, ensuring natural movement during the tasks.

Synchronization and Task Instructions: Audio instructions synchronized with the EMG signals guided participants’ movements, ensuring the consistent timing and execution of tasks. This synchronization is crucial for correlating muscle activity with the specific phases of the movements during the seated maximal reaching tasks.

#### 2.3.2. Perceived Exertion

The Borg Category Ratio-10 (CR-10) questionnaire [41] was used to assess the subjects’ perceived exertion with the CMPTO during the Reach stage. The CR-10 scale is a simple numerical rating scale ranging from 0 (rest) to 10 (maximal), with verbal descriptors associated with each number. The CR-10 was filled once all the tasks were completed in a condition. In total, each subject provided 20 RPE scores, covering five directions (0°, 45°, 90°, 135°, and 180°) with two limbs (right, left) under two different orthosis conditions (noOR/OR).

#### 2.3.3. Functionality Scales

The dystrophinopathy patients were assessed for the functionality of their upper and lower extremities using the Brooke [42] and Vignos [43] scales. The Brooke scale consists of six levels, ranging from Level 1 (indicating full range of motion and function) to Level 6 (indicating complete loss of function). The Vignos scale consists of 10 levels, ranging from Level 1 (indicating normal gait and mobility) to Level 10 (indicating confinement to a bed). Each level of these scales describes the degree of function and mobility, with specific criteria defining the abilities and limitations at each level. 

#### 2.3.4. Usability

The System Usability Scale (SUS) [44] was used to obtain feedback on the CMPTO. The questions of the SUS are designed to capture various aspects of usability, including ease of use, learnability, efficiency, and satisfaction. The SUS consists of a 10-item questionnaire, with each item scored on a 5-point Likert scale ranging from “Strongly Disagree” to “Strongly Agree”. The SUS score is found by adding up the scores for all odd number questions and deducting 5 from the total. For the even number questions, their total score is subtracted from 25. Finally, the scores of odd- and even-numbered questions are summed and multiplied by 2.5 to obtain the final SUS score.

SUS Score = [(Sum of odd Questions − 5) + (25 − Sum of even Questions)] × 2.5

### 2.4. Data Processing

**Figure 4:**
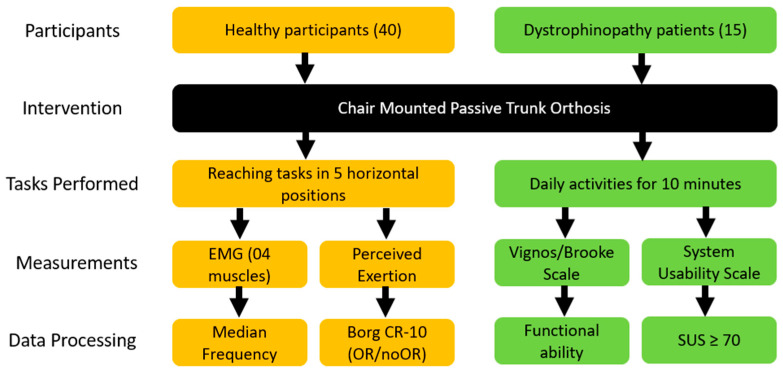
A flowchart of data processing for healthy participants and dystrophinopathy patients.

**Figure 5:**
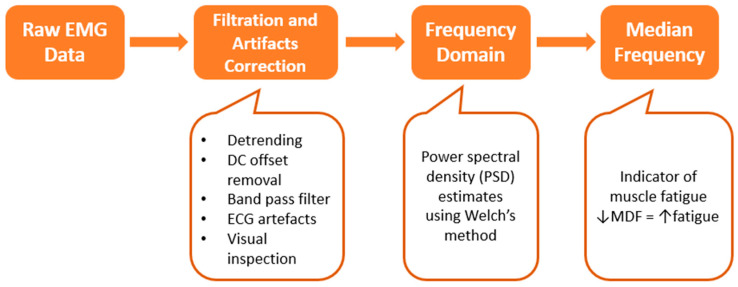
A block diagram of the steps of EMG processing.

Figure 4 presents a flowchart of data processing for both healthy participants and dystrophinopathy patients. The data processing was conducted using MATLAB 2023a (MathWorks, Natick, MA, USA), a robust software platform commonly used for signal processing and analysis. For the muscle activity data of healthy participants, several crucial pre-processing steps were necessary to ensure data accuracy and reliability. These steps, illustrated in the block diagram in Figure 5, were executed using custom-developed MATLAB code.

Initially, the raw EMG signals underwent a series of filtration and artifact correction procedures. This involved detrending the signals to remove any linear trends and eliminating the DC offset to center the signal around zero. To isolate the desired EMG signal, a band pass filter with a frequency range of 20–450 Hz was applied. This range was chosen to capture the typical frequency components of muscle activity while excluding noise and irrelevant data. Additionally, artifacts resulting from power line interference (50 Hz) and heart muscle activity were eliminated using a 50 Hz notch filter and a 30 Hz high-pass filter, respectively. These filters were designed, using the MATLAB ‘butter(n, Wn, ftype)’ command, as 3rd-order (n) bi-directional IIR Butterworth filters, known for their stable performance and minimal phase distortion. Appropriate parameters of cutoff frequency (Wn) and filter type (ftype) were set for each filter. The resulting filter transfer function coefficients (a, b) were subsequently used in the ‘filtfilt(b, a, x)’ command with the input signal (x) for zero-phase filtering.

Data analysis focused on the Reach stage due to its exhaustive nature and significance in this study. To extract the EMG signal specific to the Reach task, 4 s epochs were segmented for each trial. From these epochs, a 2 s segment, corresponding to 4096 samples and anticipated to represent a static posture, was selected for further analysis. This segment was taken from 1.5 to 3.5 s after the onset of the Reach stage, a period identified as crucial for capturing peak muscle activity. The experiment yielded a total of 80 epochs per subject, derived from 05 orientations (0, 45, 90, 135, 180 degrees) × 02 limbs (right, left) × 04 muscles (RTES, LTES, RLES, LLES) × 02 conditions (OR, noOR). Each subject performed 03 trials for each task, and the epochs from these trials were averaged to represent the corresponding EMG signal, thereby enhancing the reliability of the data.

The subsequent step in EMG data processing involved converting the signals to the frequency domain, a critical phase for understanding the spectral characteristics of muscle activity. The frequency spectrum of the epoch EMG signals was calculated by estimating their power spectral density (PSD) using the nonparametric method of Welch’s [45] overlapped segment averaging estimator with the ‘[pxx,f] = pwelch(x, window, noverlap, fr, fs)’ command in MATLAB. The input signal (x) was given along with the length of the segment window (window) for eight segments with a sliding Hamming window, the number of overlapping segments (noverlap) as 50%, desired 4096 frequencies (fr), and a sampling rate (fs) of 2048 Hz. This method, known for its effectiveness in reducing variance, improved estimation quality by controlling spectral leakage and variance. The resulting two-sided PSD estimate (pxx) and cyclical frequencies (f) were then used to determine the median frequency in the epochs for all tasks using the MATLAB command ‘medfreq(pxx,f)’. The median frequency, which divides a PSD into two parts of equal energy, was calculated using the following formula [46] that accounts for the energy distribution within the signal.

$$\int_{f1}^{Fmedian}PS\left(f\right)\cdot df=\int_{Fmedian}^{f2}PS\left(f\right)\cdot df$$

where *PS*(*f*) is the PSD estimate, *f*1 is the lowest EMG frequency in the bandwidth, and *f*2 is the highest EMG frequency in the bandwidth. The code for pre-processing of EMG data is provided in Supplementary Materials.

Changes in the EMG median frequency were used as indicators of muscle fatigue, a key parameter in understanding muscle performance and endurance. Additionally, subjective measurements of the rate of perceived exertion (RPE), which defines exertion levels during tasks, were recorded from healthy subjects to correlate physical performance with perceived effort. For the dystrophinopathy patients, the Brooke and Vignos scale scores, which define the level of functional ability, were noted to provide a comprehensive understanding of their physical capabilities. The System Usability Scale (SUS) questionnaire scores were normalized and combined to produce a single usability score ranging from 0 to 100, with a score of ≥70 considered acceptable. This comprehensive analysis aimed to provide a holistic understanding of muscle activity, fatigue, and usability in both healthy participants and dystrophinopathy patients.

### 2.5. Statistical Analysis

The 4-way ANOVA was applied for the dependent variable of median frequency where the independent variables were Orthosis, Muscle, Limb Used, and Task Orientation. The main effects, as well as interaction effects, were found, and the *p*-values for each comparison were corrected for the family-wise error rate due to multiple comparisons using the Bonferroni–Holm correction method. The effect sizes (eta-squared η^2^) were computed for each effect in the ANOVA as the ratio between the effect’s sum of squares and the total sum of squares. Paired *t*-tests were applied between the median frequency found in OR and noOR conditions for all epochs. The ratings of perceived exertion in each task under both conditions were analyzed using the nonparametric Wilcoxon signed-rank test to identify any statistically significant variances. The Mann–Whitney U test was used to compare the SUS score. The significance level alpha was kept at 0.05 for all statistical tests.

## 3. Results

### 3.1. Variance in Median Frequency

Table 1 presents the summarized ANOVA results from the Reach stage. The individual and interaction effects of sources are represented row-wise. The degrees of freedom for each effect are given together with the F-statistic, *p*-value corrected for multiple comparisons, and eta^2^ for the effect sizes. The main effects of ‘Task Orientation’ (F = 8.33, *p* < 0.001, η^2^ = 0.009), ‘Muscle’ (F = 39.01, *p* < 0.001, η^2^ = 0.031), and ‘Orthosis’ (F = 7.33, *p* = 0.048, η^2^ = 0.002) were found to be significant, while that of ‘Limb Used’ was not significant. The significant two-interaction effects were observed for ‘Task Orientation × Limb Used’ (F = 4.42, *p* = 0.015, η^2^ = 0.005), ‘Task Orientation × Muscle’ (F = 17.65, *p* < 0.001, η^2^ = 0.055), and ‘Muscle × Orthosis’ (F = 4.85, *p* = 0.020, η^2^ = 0.004). In three-interaction effects, the ‘Task Orientation × Limb Used × Muscle’ (F = 5.39, *p* < 0.001, η^2^ = 0.017), ‘Task Orientation × Limb Used × Orthosis’ (F = 3.91, *p* = 0.029, η^2^ = 0.004), and ‘Limb Used × Muscle × Orthosis’ (F = 5.62, *p* = 0.008, η^2^ = 0.005) were significant. The highest effect size was found for the ‘Task Orientation × Muscle’ (ƞ^2^ = 0.055) effect, followed by the ‘Muscle’ (ƞ^2^ = 0.031) effect, and then the ‘Task Orientation × Limb Used × Muscle’ (ƞ^2^ = 0.017) effect.

**Figure 6:**
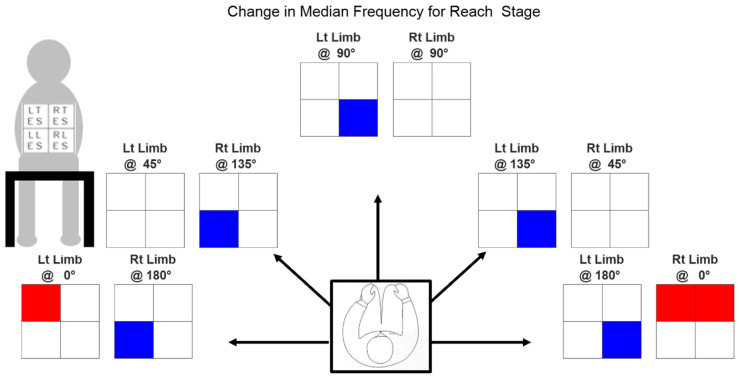
The Reach stage significantly changes (shaded) in the median frequency of the electromyographic data due to the CMPTO. The ten subplots represent the 10 tasks, i.e., at 5 orientations (0°, 45°, 90°, 135°, and 180°) using 2 limbs (Rt: right, Lt: left). Within each subplot, the four boxes represent LTES, RTES, LLES, and RLES muscles, as indicated by the top-left representation. The red shade represents a significant increase, while the blue shade represents a significant decrease in the median frequency (*p* = 0.05).

Table 2 provides the changes in median frequencies in the OR condition with respect to noOR condition, observed for each muscle across all orientations with either limb. Figure 6 summarizes the significant changes among these observations. The figure displays ten heatmaps, with each heatmap divided into four boxes representing the average change in median frequency for individual muscles between the two conditions: OR and noOR. These changes are depicted across each limb and task orientation. The rows in the heatmap represent the muscle groups, specifically TES muscles for the first row and LES muscles for the second row. The columns in the heatmap depict the lateral sides as viewed from behind, with the left side represented in the first column and the right side in the second column. The individual cells of the heatmap can be either unshaded, shaded blue, or shaded red. A red cell indicates a significant positive change in median frequency, whereas a blue cell indicates a significant negative change in median frequency when using the CMPTO. The cells that are unshaded represent a non-significant change in median frequency.

A notable reduction in the median frequency of the ipsilateral LES muscle occurs when utilizing the CMPTO to execute horizontally adducted movements, such as moving towards the left side with the right limb and towards the right side with the left limb. For the RLES muscle, reductions are observed with the left limb at 90° (−5.327 Hz), 135° (−2.982 Hz), and 180° (−0.670 Hz). For the LLES muscle, reductions are observed with the right limb at 135° (−10.860 Hz) and 180° (−10.405 Hz). Moreover, we see that TES muscles show an increase in the median frequency when the CMPTO is used for making movement at 0°, with the right limb (RTES: 2.279 Hz, LTES: 3.326 Hz) and the left limb (LTES: 3.170 Hz).

### 3.2. Ratings of Perceived Exertion

**Figure 7:**
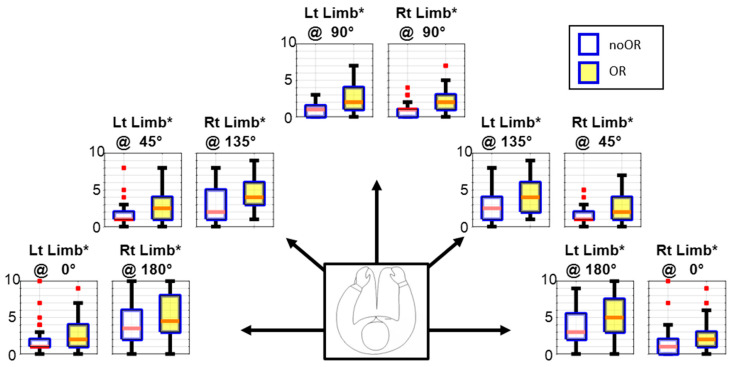
The ratings of perceived exertion (RPE) using the Borg CR-10 scale for noOR and OR conditions. The ten box plots represent the ten tasks, i.e., at five orientations (0°, 45°, 90°, 135°, and 180°) using two limbs (Rt: right, Lt: left). The red dots represent the outliers. An asterisk in the subplot title indicates a significant difference in the RPE between both conditions (*p* = 0.05).

Figure 7 depicts the median RPE ratings on the Borg CR-10 scale during the Reach stage. The figure has ten box plots representing five orientations and two limbs. In each box plot, there are two boxes shaded yellow and white for OR and noOR conditions, respectively. The red line inside the box represents the middle value of the data, i.e., the median. The red dots show the outliers, whereas the black lines show the interquartile range (IQR). Asterisks in box plot titles indicate a significant difference in ratings between the OR and noOR conditions. In all twenty (20) tasks, the RPE for the noOR condition is significantly greater. The RPEs for horizontally abducted upper limb orientations are lower than those for horizontally adducted upper limb orientations (0°, 45° vs. 135°, 180°). There are more outliers in data for horizontally abducted upper limb orientations, whereas the IQR is greater for the horizontally adducted upper limb orientations. At 90° orientation, the ratings with either upper limb are small as well as comparable.

### 3.3. Usability Ratings by Patients

Table 3 presents the patient demographics and their scores for the Brooke, Vignos, and SUS. The patients involved in the feedback session were aged 24.93 ± 11.63 (mean ± sd) years old. Their lower limb functional abilities were predominantly situated toward the lower end of the spectrum, as indicated by the Vignos scores (mean ± sd: 8.93 ± 0.59) ranging from 7 to 10. The Brooke scores (mean ± sd: 4.57 ± 0.89) ranged from 2 to 6, indicating varying degrees of upper extremity impairment. Notably, despite the challenges posed by their condition, all participants rated an acceptable usability with scores ≥70. The SUS scores obtained from DMD patients (mean = 84.3) were significantly (*p* = 0.0360) higher than those obtained from healthy subjects (mean = 75.5).

## 4. Discussion

The present study investigated the impact of the CMPTO on psychophysiological manifestations of fatigue, with a particular focus on its suitability for dystrophinopathy patients. Seated reaching tasks were performed along different orientations to represent the 3-dimensional trunk motion involving trunk flexion, lateral bending, and rotation, as required when performing the activities of daily living. This study analyzed the muscle activity and perceived exertion during these tasks, as well as the feedback obtained from patients. The results of this study provide valuable insights into the potential benefits and safety of the CMPTO as an assistive device for individuals with dystrophinopathy.

### 4.1. Manifestations of Fatigue

The RPE was used as an indicator of fatigue [47] during maximal reaching isometric tasks [48]. The RPE was found to be higher with the CMPTO, which is a consistent finding with assistive devices for dystrophinopathy patients [17] and contrasts with assistive devices for healthy persons [27,29]. The participants were only required to overcome their body’s inertia and air resistance in the noOR condition, whereas, when utilizing the CMPTO, the maximal stretching towards the target involved overcoming the additional resistance provided by the spring force. Hence, the exertion was higher. On the contrary, for assistive devices for healthy persons, the purpose was to transfer the trunk muscle load to other places on the body. Therefore, although trunk muscles became relaxed, the overall work conducted was in fact greater as the device had to be worn with additional weight. Nevertheless, the added resistance effect, in our case, may have potential benefits for resistance training in neuromuscular rehabilitation where increasing physical exertion is desirable [49,50]. Additionally, the stored applied energy in CMPTO springs could potentially assist in the returning phase of movement. 

Interestingly, the tasks involving horizontally adducted arm movements were found to be perceived as challenging due to the increased trunk rotation [51] requirement, demanding greater effort from individuals. The RPEs for these tasks displayed considerable variability among subjects, suggesting that some individuals found the task relatively easy, as has also been reported with arm support assistive devices [17]. This variability may imply that achieving the same postural change can be accomplished across a wide range of RPE scores [52]. Moreover, the RPE in this study represents the maximum exertion possible, whereas most daily activities typically require lower levels of intensity [53]. Therefore, we expect low exertion with the CMPTO in daily use. Nevertheless, the RPE for both conditions shows minimal difference, suggesting that the CMPTO imposes only a slight strain on the user.

It is intriguing to note that the median frequency of each muscle’s EMG signal varies distinctly according to the task orientation. Furthermore, the use of the CMPTO during seated reaching tasks did not result in a predominantly significant decrease in the median frequency. This finding is particularly noteworthy because a decrease in the median frequency is indicative of muscle fatigue [23], which is a common concern for individuals with dystrophinopathy due to their progressive muscle weakness and limited mobility [54]. Maintaining a static posture without experiencing excessive fatigue is crucial for enhancing the quality of life and functional independence of these patients [10]. The fact that the CMPTO did not exacerbate fatigue suggests that it may be a well-tolerated assistive device, especially during prolonged periods of sitting. This finding is consistent with previous studies where the use of assistive devices led to a decrease in fatigue [17,18,19]. However, we did observe a decrease in the median frequency during tasks perceived as the most demanding by the subjects. These tasks involved moving the upper limb to the contralateral side. Although included in the experimental protocol, this movement may seem counterintuitive to the patients, who may typically utilize their ipsilateral upper limb for daily activities [55].

Moreover, we observed a few instances of increased median frequency at the 0° orientations with both limbs. This could potentially be attributed to a misalignment of the harness [56]. The subjects were seated on an experimental chair with greater than usual depth, to evade the effect of back support. As they attempted movements at 0° orientations, they may have inadvertently shifted backward, causing the harness to loosen over the shoulders. Consequently, the spring force did not effectively transfer to the trunk, and no counter muscle force was required. It is worth noting that when the CMPTO is fitted on a wheelchair, such loosening cannot occur as the patient’s trunk would be in contact with the wheelchair’s backrest.

### 4.2. Perspectives of Patients

The patients found the CMPTO to be more user-friendly compared to healthy subjects, likely attributed to their deeper understanding of the disease’s complexities and specific needs. Most DMD patients lose ambulation before the age of 13 years [8]. Consequently, given the progressive nature of the disease, it is likely that around this age, there is a decline in trunk muscle strength, necessitating the use of trunk assistive devices. Hence, it seems that the CMPTO offers the most advantages for patients aged 14–15 years and those with functional capability scores of Brooke: 5 and Vignos: 9. Studies with similar age groups of patients [19] have also indicated positive outcomes with orthosis, suggesting that the lower SUS ratings observed in young DMD patients and BMD patients may be attributed to the slower progression of their disease [2]. This slower progression could lead to the lack of anticipation regarding the forthcoming challenges in trunk mobility [57], causing them to underestimate the benefit of the CMPTO.

### 4.3. Suggestions for CMPTO Usage

Our previous study provided details about the design of the CMPTO [35]. Moving forward, this study focused on the evaluation of the CMPTO during its usage by dystrophinopathy patients during the activities of daily living. The orthosis provides essential support to maintain an upright sitting posture which is crucial for individuals with weakened trunk muscles due to dystrophinopathy [30]. By promoting proper posture and trunk alignment, the CMPTO can aid in improving respiratory function by expanding the chest cavity, making breathing easier and more efficient for patients with compromised respiratory muscles [58]. The integration of the CMPTO in wheelchairs may enhance mobility, ensuring stability while moving and allowing for greater independence in performing daily activities.

The CMPTO may also help in distributing weight evenly across the trunk, reducing the risk of pressure sores and discomfort for patients who spend prolonged periods seated. This is beneficial for preventing skin breakdown and enhancing overall comfort [59]. Moreover, proper trunk support enables patients to engage better in daily activities such as eating, using a computer, or participating in social interactions, facilitating more engagement in social life [34]. Furthermore, during physical therapy sessions, the CMPTO may provide stable support, making it easier to perform exercises aimed at maintaining muscle strength and flexibility, thus reducing the risk of further injury during therapeutic activities [60].

For younger patients attending school or adults involved in work, the CMPTO can assist with maintaining proper posture for extended periods, enhancing their ability to participate effectively in educational and professional environments [61]. Proper trunk support can also boost confidence and reduce anxiety related to posture and mobility challenges, contributing to improved psychological well-being [62]. Therefore, the use of the CMPTO may address both the physical and psychological needs of dystrophinopathy patients.

### 4.4. Limitations and Recommendations

It is essential to acknowledge certain limitations and considerations in this study. First, the muscle activity data from dystrophinopathy patients could not be collected. This limitation arose due to the potential complexities introduced by variations in muscular contraction mechanisms resulting from spinal deformities in these patients, which could have complicated the interpretation of the muscle activity results [63]. Second, this study primarily assessed muscle fatigue during static postures for limited orientations in the horizontal seated plane. While this was essential to generalize the trunk motion, it is equally important to investigate the impact of the CMPTO during the dynamic activities and functional tasks of patients. Future studies could explore the CMPTO’s effects in a broader range of daily life activities. Additionally, while the muscles under scrutiny in this study, namely the erector spinae muscles, hold pivotal significance in seated balance [64], future studies could examine more muscles of the trunk for exploring the global effect of the CMPTO. Lastly, the CMPTO can be advanced by incorporating movement intention detection with machine learning [65] and neurofeedback [66] technologies, to provide the user with a more intuitive experience. Moreover, as the prevalence of mental health issues has doubled [67], future studies should take these issues into account when examining the subjects for the potential acceptability of the CMPTO. This consideration may introduce a novel dimension to evaluate psychometric alterations induced during the use of the trunk orthosis due to current [68], thermal [69], or auditory [70,71] stimulation.

## 5. Conclusions

This study offers promising evidence suggesting that the CMPTO is a safe assistive device for dystrophinopathy patients. It notably did not induce significant strain on users during moderately exhaustive tasks. This study also identifies the disease stage at which the CMPTO is the most effective and provides suggestions for its usage. Overall, the CMPTO demonstrates potential as a valuable tool for improving the comfort and functional abilities of individuals living with dystrophinopathies.

## Figures and Tables

**Table 1:** Summary of median frequency ANOVA results regarding main and interaction effects of Task Orientation, Limb Used, Muscle, and Orthosis factors on median frequency. F-statistic, *p*-value, and effect size are given, and statistically significant results are bold-typed.

Source	df	F	*p*	η^2^
**Task Orientation**	**4**	**8.33**	**<0.001**	**0.009**
Limb Used	1	5.57	0.110	0.002
**Muscle**	**3**	**39.01**	**<0.001**	**0.031**
**Orthosis**	**1**	**7.33**	**0.048**	**0.002**
**Task Orientation** × **Limb Used**	**4**	**4.42**	**0.015**	**0.005**
**Task Orientation** × **Muscle**	**12**	**17.65**	**<0.001**	**0.055**
Task Orientation × Orthosis	4	0.11	1.000	0.000
Limb Used × Muscle	3	3.14	0.122	0.003
Limb Used × Orthosis	1	0.30	1.000	0.000
**Muscle** × **Orthosis**	**3**	**4.85**	**0.020**	**0.004**
**Task Orientation** × **Limb Used** × **Muscle**	**12**	**5.39**	**<0.001**	**0.017**
**Task Orientation** × **Limb Used** × **Orthosis**	**4**	**3.91**	**0.029**	**0.004**
Task Orientation × Muscle × Orthosis	12	1.60	0.336	0.005
**Limb Used** × **Muscle** × **Orthosis**	**3**	**5.62**	**0.008**	**0.005**
Task Orientation × Limb Used × Muscle × Orthosis	12	0.62	1.000	0.002

**Table 2:** Change in median frequency of EMG signal of each muscle at all orientations, using upper limbs. Bold font represents significant values. Abbreviations—RTES: Right Thoracic Erector Spinae, LTES: Left Thoracic Erector Spinae, RLES: Right Lumbar Erector Spinae, LLES: Left Lumbar Erector Spinae, Ex: Extend stage, Re: Reach stage, Rt: right limb, Lt: left limb.

Muscle Orientation			RTES	LTES	RLES	LLES
Limb Used	Rt	0°	2.279	3.326	−0.133	1.564
		45°	0.854	−0.553	−3.013	2.209
		90°	−0.443	−2.142	−0.109	−1.947
		135°	2.516	−1.874	−2.020	−10.860
		180°	0.476	−1.705	0.153	−10.405
	Lt	0°	1.325	3.170	−0.670	1.458
		45°	1.455	0.715	−2.982	2.747
		90°	−1.400	0.417	−5.327	−3.584
		135°	1.067	−0.765	−13.333	1.857
		180°	−2.502	−0.971	−16.714	1.222

**Table 3:** Patient demographics and SUS scores.

Subject	Age	Dystrophy	Brooke	Vignos	SUS Score
S1	06	Duchenne	2	7	70.0
S2	14	Duchenne	5	9	97.5
S3	14	Duchenne	6	10	90.0
S4	15	Duchenne	5	9	92.5
S5	18	Becker	4	9	–
S6	19	Duchenne	5	9	–
S7	21	Becker	4	9	–
S8	23	Becker	4	9	87.5
S9	27	Duchenne	5	9	–
S10	28	Becker	5	9	80.0
S11	29	Becker	5	9	–
S12	30	Becker	5	9	–
S13	40	Becker	5	9	82.5
S14	43	Becker	5	9	75.0
S15	47	Becker	5	9	–

## Data Availability

The data supporting this study’s findings are available upon request from the corresponding authors.

## Supplementary Materials

The following supporting information can be downloaded at: https://www.mdpi.com/article/10.3390/bioengineering11080780/s1, Code S1: Pre-processing code.
